# Supplementary material for: Evaluating the Risk of Inguinal Lymph Node Metastases before Surgery Using the Morphonode Predictive Model: A Prospective Diagnostic Study in Vulvar Cancer Patients
Source: Cancers (Basel). 2023 Feb 9;15(4):1121. doi: 10.3390/cancers15041121 (PMC9953890; doi:10.3390/cancers15041121)
Supplement: Supplementary file 1 [file cancers-15-01121-s001.zip › cancers-2175717-supplementary.pdf]

Supplementary Table S1. Ultrasound parameters of the 237 groins.

**Characteristic [a]**	**All (n = 237)**	**N0 - Negative lymph node at histology (n = 162)**	**N1 - Positive lymph node at histology (n = 75)**	**Test [b]**	**Estimate [c]**	**CI 95% [d]**	**P-value**
**Dimensional parameters**							
Long axis (mm)	18.0 (2.8-57.0)	17.6 (2.8-54.0)	18.0 (6.4-57.0)	wilcoxon	2	-0.2, 4.2	0.082
Short axis (mm)	**7.0 (1.1-30.0)**	**6.2 (1.1-20.0)**	**10.0 (4.0-30.0)**	wilcoxon	**3,1**	**2.3, 4.0**	**< 0.001**
Long/Short axis ratio	**2.5 (1.0-11.0)**	**2.7 (1.0-11.0)**	**1.9 (1.1-7.9)**	wilcoxon	**-0,7**	**-1.0, -0.4**	**< 0.001**
< 2	**74 (31%)**	**35**	**39**	proportion	**0.520, 0.216**	**0.165, 0.443**	**< 0.001**
>= 2	**163 (69%)**	**127**	**36**	proportion	**0.480, 0.784**	**-0.443, -0.165**	**< 0.001**
Cortical thickness (mm)	**3.0 (0.1-44.0)**	**2.5 (0.1-44.0)**	**4.5 (1.7-37.0)**	wilcoxon	**1,9**	**1.4, 2.4**	**< 0.001**
Medulla thickness (mm)	**3.4 (0.3-52.0)**	**3.1 (0.3-52.0)**	**4.0 (1.2-29.8)**	wilcoxon	**0,6**	**0.1, 1.0**	**0.006**
Cortex/Medulla thickness ratio	**0.9 (0.1-10.8)**	**0.8 (0.1-6.2)**	**1.3 (0.4-10.8)**	wilcoxon	**0,4**	**0.2, 0.6**	**< 0.001**
<= 1	**131 (55%)**	**104**	**27**	proportion	**0.360, 0.642**	**-0.423, -0.141**	**< 0.001**
> 1	**106 (45%)**	**58**	**48**	proportion	**0.640, 0.358**	**0.141, 0.423**	**< 0.001**
**Morphological parameters**							
Perinodal hyperecogenic ring	**29 (12%)**	**3**	**26**	proportion	**0.347, 0.019**	**0.209, 0.448**	**< 0.001**
Cortical interruption	**19 (8%)**	**2**	**17**	proportion	**0.227, 0.012**	**0.108, 0.320**	**< 0.001**
Nodal core sign absence	**29 (12%)**	**4**	**25**	proportion	**0.387, 0.025**	**0.239, 0.485**	**< 0.001**
Cortical thickening							
Absent	**98 (41.4%)**	**87**	**11**	proportion	**0.147, 0.537**	**-0.511, -0.270**	**< 0.001**
Focal	39 (16.4%)	28	11	proportion	0.147, 0.173	-0.135, 0.083	0.751
Concentric	**45 (19%)**	**25**	**20**	proportion	**0.267, 0.154**	**-0.012, 0.237**	**0.061**
Eccentric	**37 (15.6%)**	**20**	**17**	proportion	**0.227, 0.123**	**-0.014, 0.220**	**0.065**
Not evaluable	18 (7.6%)	2	16	---	---	---	---
Ecogenicity							
homogeneous	**147 (62%)**	**122**	**25**	proportion	**0.333, 0.753**	**-0.555, -0.284**	**< 0.001**

Inhomogeneous	**90 (38%)**	**40**	**50**	proportion	**0.667, 0.247**	**0.284, 0.555**	**< 0.001**
Diffuse pattern	**56 (23.6%)**	**18**	**38**	proportion	**0.507, 0.111**	**0.263, 0.528**	**< 0.001**
Focal intranodal deposit	16 (6.8%)	12	4	proportion	0.053, 0.074	-0.095, 0.054	0.754
Type of deposit							
Hyperechoic	44 (18%)	25	19	proportion	0.253, 0.154	-0.024, 0.222	0.100
Anaechoic (cystic areas)	7 (3%)	3	4	proportion	0.053, 0.019	-0.030, 0.100	0.289
Both	**21 (9%)**	**2**	**19**	proportion	**0.253, 0.012**	**0.131, 0.351**	**< 0.001**
Cortex/Medulla interface distortion							
Absent	**141 (59.5%)**	**116**	**25**	proportion	**0.333, 0.716**	**-0.520, -0.246**	**< 0.001**
Present	**79 (33.3%)**	**44**	**35**	proportion	**0.467, 0.272**	**0.053, 0.337**	**0.005**
Focal	43 (18%)	26	17	proportion	0.227, 0.160	-0.054, 0.186	0.295
Diffused	**36 (15%)**	**18**	**18**	proportion	**0.240, 0.111**	**0.011, 0.247**	**0.017**
Not visible	**17 (7%)**	**2**	**15**	proportion	**0.200, 0.012**	**0.086, 0.290**	**< 0.001**
Shape							
Elliptical (oval)	**160 (67.5%)**	**125**	**35**	proportion	**0.467, 0.772**	**-0.445, -0.165**	**< 0.001**
Round	**40 (16.9%)**	**20**	**20**	proportion	**0.267, 0.123**	**0.021, 0.265**	**0.011**
Irregular (lobulated/spiculated)	**37 (15.6%)**	**17**	**20**	proportion	**0.267, 0.105**	**0.041, 0.282**	**0.003**
Grouping							
Absent	175 (74%)	125	50	proportion	0.667, 0.772	-0.239, 0.030	0.121
Partial (moderate)	48 (20%)	34	14	proportion	0.187, 0.210	-0.141, 0.095	0.811
Complete	**14 (6%)**	**3**	**11**	proportion	**0.013, 0.049**	**0.036, 0.221**	**< 0.001**
**Dominant vascular flow architecture**							
Pattern							
Not vascularized	10 (4.2%)	8	2	proportion	0.027, 0.049	-0.082, 0.036	0.644
Longitudinal axis	**123 (51.9%)**	**103**	**20**	proportion	**0.267, 0.636**	**-0.503, -0.235**	**< 0.001**
Scattered	35 (14.8%)	18	17	proportion	0.227, 0.111	-0.001, 0.232	0.033
Branched	**53 (22.4%)**	**27**	**26**	proportion	**0.347, 0.167**	**0.048, 0.312**	**0.003**

Chaotic	**16 (6.7%)**	**6**	**10**	proportion	**0.133, 0.037**	**0.004, 0.188**	**0.014**
Localization							
Not vascularized	9 (3.8%)	8	1	proportion	0.013, 0.049	-0.088, 0.016	0.325
Central (hilar)	**159 (67%)**	**126**	**33**	proportion	**0.440, 0.778**	**-0.477, -0.199**	**< 0.001**
Transcapsular	**22 (9.3%)**	**4**	**18**	proportion	**0.240, 0.025**	**0.106, 0.325**	**< 0.001**
Peripheral (cortical)	**8 (3.3%)**	**0**	**8**	proportion	**0.107, 0.000**	**0.027, 0.186**	**< 0.001**
Extranodal	**14 (6%)**	**4**	**10**	proportion	**0.133, 0.025**	**0.018, 0.199**	**0.003**
Combined	**47 (19.8%)**	**24**	**23**	proportion	**0.307, 0.148**	**0.031, 0.286**	**0.008**
**Color score**							
1	**101 (42.6%)**	**82**	**19**	proportion	**0.253, 0.506**	**-0.388, -0.118**	**< 0.001**
2	83 (35%)	57	26	proportion	0.347, 0.352	-0.141, 0.130	0.999
3	**47 (19.8%)**	**21**	**26**	proportion	**0.347, 0.130**	**0.088, 0.346**	**< 0.001**
4	6 (2.6%)	2	4	proportion	0.053, 0.012	-0.022, 0.104	0.155
Morphometric ultrasound pattern (subjective assessment)							
LN1	**66 (27.8%)**	**61**	**5**	Fisher's exact	**17,685**	**7.960, 43.765**	**< 0.001**
LN2	**58 (24.5%)**	**54**	**4**				
LN 3	**58 (24.5%)**	**37**	**21**				
LN 4	**20 (8.4%)**	**9**	**11**				
LN 5	**35 (14.8%)**	**1**	**34**				
**Fine-needle aspiration cytology**	92 (39%)	48	44	proportion	**0.587, 0.296**	**0.149, 0.432**	**< 0.001**
Cytology/Histology report							
C1	**0 (0%)**	**0**	**0**	Fisher's exact	**31,838**	**6.852, 301.706**	**< 0.001**
C2	**64 (27%)**	**46**	**18**				
C3	**1 (0.5%)**	**1**	**0**				
C4	**1 (0.5%)**	**1**	**0**				
C5	**26 (11%)**	**0**	**26**				
**Final Overall Assessment (FOA)**							
Positive	**72 (30.4%)**	**18**	**54**	Fisher's exact	**20,17**	**9.659, 44.264**	**< 0.001**
Negative	**165 (69.6%)**	**144**	**21**				

Results are present as number (%) or median (range). Ultrasound parameters were assessed on the dominant lymph node.

[a] Median (range) for continuous variables, number of occurrences (percentage) for categorical variables

[b] Two-sided Wilcoxon's rank sum test for continuous variables;
Two-sided test for equality of proportions for count variables;
Two-sided Fisher's exact test for surrogate variables of the primary outcome.

[c] Distribution shift estimate for Wilcoxon's rank sum tests;
Proportions for positive and negative lymph nodes for proportions tests;
Odds ratio for Fisher's exact tests.

[d] 95% confidence interval for:
Shift estimate (Wilcoxon's rank sum test);
positive-negative proportion difference (proportions equivalence test);
odds ratio (Fisher's exact test).
